# Exercise Testing for Metabolic Flexibility: Time for Protocol Standardization

**DOI:** 10.1186/s40798-025-00825-w

**Published:** 2025-03-31

**Authors:** Dale I. Lovell, Max Stuelcken, Alexander Eagles

**Affiliations:** 1 School of Health, The University of the Sunshine Coast, Maroochydore, QLD 4556 Australia; 2 Timeless Medical Australia, Maroochydore, QLD 4556 Australia

**Keywords:** Metabolic Flexibility, Exercise, Protocol, Lactate, Screening, Health

## Abstract

Metabolic syndrome (MetS) is a combination of risk factors that contribute to the development of many of today’s chronic diseases. Rates of MetS continue to increase and it is now considered a worldwide epidemic. As with many chronic diseases it may take years for symptoms and the effects of MetS to manifest into severe health problems. Therefore, early detection is paramount A recently proposed method for the early detection of MetS is the assessment of an individual’s metabolic flexibility during exercise. Metabolic flexibility is defined as the ability of the body to switch between energy substrates, primarily fats and carbohydrates, to produce energy and meet metabolic demand. This provides an indication of mitochondrial health, the possible beginning point of early insulin resistance and the development of MetS.

Although there is widespread use of exercise and expired gas analysis to determine metabolic flexibility, there is no consensus on the appropriate guidelines, protocol, or interpretation of the subsequent data. Studies have used a variety of different protocols involving maximal and submaximal tests with step protocols ranging from 2 to 10 min, differences in data averaging, analysis, and stoichiometric equations, as well as variations in nutritional status of participants, and mode of exercise. This has led to considerable variation in reported results. Although the use of exercise to determine metabolic flexibility and act as a possible marker of early mitochondrial dysfunction holds significant promise, more work is required to determine the optimal protocol for clinical and research purposes.

## Key Points

Metabolic flexibility is the ability of skeletal muscle to adjust its utilization of substrate pathways to meet metabolic demand and is an indication of mitochondrial health.

Exercise provides the ideal stimulus to determine metabolic flexibility. However, the absence of a universal protocol has resulted in many different methods and consequently a large variation in results.

Further work is required to establish a standardized exercise test for metabolic flexibility. Such a test may provide an easy, time efficient, relatively non-invasive measure of metabolic flexibility which can be administered by health care providers for the assessment of mitochondrial health.

## Introduction

Chronic disease (CD) includes non-communicable diseases such as cardiovascular disease (CVD), metabolic syndrome, neurological and respiratory disease, and cancer. Although around 80% of CVD and metabolic syndrome may be preventable by reducing/eliminating four lifestyle risk factors - smoking, poor nutrition, alcohol misuse and physical inactivity [1], the rates of CVD have been increasing in younger populations (< 55 years of age) in more recent years [2, 3]. This may, in part, be due to the considerable rise in metabolic syndrome (MetS) which has become a global epidemic [4, 5].

## Metabolic Syndrome

Although there is some debate over the exact definition of MetS [5], it is a pathological condition characterized by the presence of any three of the following: abdominal obesity, insulin resistance, hypertension, and hyperlipidaemia [4]. (For a more detailed definition see [5]). What is clear, is the strong scientific evidence linking MetS with increased rates of other CDs such as CVD [6], Type 2 diabetes [7], kidney disease [8], non-alcoholic fatty liver disease [9], cancer [10], and various neurological diseases [11].

Nevertheless, a better understanding of the pathophysiological mechanism linking MetS and CDs is needed. This would allow a more targeted approach for interventions to combat the rise in MetS. Whilst genetics and environmental factors [12] may have some role in MetS, recent evidence indicates that insulin resistance (IR) may be a more important contributor to the worldwide rise in MetS [13–15]. During his Banting Lecture to the American Diabetes Association in 1988 [15], Gerald Reaven proposed that the most common cause of CVD was not an increased concentration of cholesterol but a group of risk factors (MetS listed above) resulting from a state of insulin resistance (IR). Furthermore, Reaven and colleagues established that IR was key in the aetiology of type 2 Diabetes mellitus and not insulin deficiency as previously thought [16]. Others [13, 14, 17, 18] have also confirmed these findings and identified possible mechanisms linking IR with MetS and the associated CDs.

### Insulin Resistance

Insulin production stimulates the storage of nutrients in skeletal muscle, the liver and white adipose tissue. The liver, in particular plays a prominent role in the development of IR as the liver preferentially disposes of more than 50% of post prandial glucose [19]. If the liver is constantly overwhelmed by increased amounts of CHO, the spillover to muscle will be higher resulting in increased levels of insulin and the possible development of IR [20]. Insulin resistance is also associated with an increase in ectopic lipid droplets with fatty acid metabolites such as ceramides and sn-1,2 diacylglycerols (DAGs) which inhibit insulin signalling [13, 21, 22]. In particular, DAGs within the plasma membrane of both skeletal muscle and the liver are now seen as an important factor in mediating liver and muscle IR [18, 23]. The most prominent contributor leading to increased intracellular DAGs is overnutrition. This increases fatty acid delivery to the tissues, exceeding mitochondrial fat oxidation and promoting triglyceride synthesis and storage. Low rates of mitochondrial fat oxidation have also been shown to contribute to intramyocellular lipid content and muscle IR in healthy, lean, young (parents with Type 2 diabetes) [24] and older individuals [25, 26] and after periods of prolonged muscle inactivity [27].

## Mitochondrial Function and Chronic Disease

Mitochondrial function has a central role in maintaining health and preventing disease [28]. Whether mitochondrial dysfunction is the primary cause or the consequence of factors such as caloric intake, physical inactivity, obesity and/or genetics is unclear. What is clearly evident is the link between the mitochondria and CDs. Mitochondrial dysfunction (MD) has been shown to precede IR and hepatic stenosis [29] and can be used as a mechanistic biomarker of non-alcoholic fatty liver disease [30]. Other CDs strongly linked to MD include CVD [31], muscle atrophy [27], kidney disease [32], neurodegenerative disease [33], atherosclerosis [34], and Type 2 diabetes [35]. MD may also provide insights into human aging [36].

### Mitochondria and Substrate Utilization

The primary function of the mitochondria is to use nutrients to provide energy for cellular activity through the process of cellular respiration. Although the bioenergetics of cellular respiration are complex, the capacity of the mitochondria to switch between energy substrates, primarily fats and carbohydrates (CHO), to produce ATP and meet metabolic requirements is defined as metabolic flexibility (MF). A lack of MF has recently been acknowledged as an underlying mechanism for MD and therefore a significant contributor to many of today’s CDs [37–40]. Shulman and colleagues [9, 13, 14] have reported that the first step in the origins of IR may be due to intramuscular lipid content and the previously mentioned DAGs. The DAGs compromise the ability of GLUT 4 transporters located within skeletal muscle to translocate to the cell surface and initiate glucose uptake into the cell. As a result, a greater concentration of insulin is required to stimulate glucose uptake [14]. Over time this leads to IR. It is therefore imperative to implement strategies to help reduce IR and MD.

### Exercise as a Method of Assessing Mitochondrial Function

To date, regular exercise has proven to be the best physiological stimulus for improving mitochondrial function and reversing IR in skeletal muscle. Exercise stimulates an increase in mitochondrial protein [41, 42], including enzymes involved in β-oxidation, the Krebs cycle and the electron transport system [28, 42, 43]. Furthermore, increases in skeletal muscle oxidative capacity also occur in response to exercise training [28]. These changes are in part, due to increases of up to 40% in mitochondrial density in response to regular endurance exercise [28, 44]. As a result of the improvements in metabolic function, regular exercise has consistently been shown to have a profound effect on IR and many of today’s CDs [15, 28, 45–48]. In contrast, a lack of adequate physical activity and/or sedentary behaviour is now recognised as the primary cause of most chronic diseases [49, 50].

Such is the importance of physical activity in delaying/preventing CDs and thereby reducing health care costs [51, 52] that there are calls for cardiorespiratory fitness to become a metric similar to other predictors of morbidity and mortality such as smoking, cholesterol, hypertension, and type 2 diabetes [53, 54]. Traditionally a maximal oxygen uptake (VO_2_ max) test is used to assess cardiorespiratory fitness and metabolic function; however, a VO_2_ max test and subsequent exercise prescription may not necessarily be the best metric for interventions targeting mitochondrial function [28, 55] and improving MF [56].

## Metabolic Flexibility

There is now a growing body of evidence supporting MF as a method of determining mitochondrial health and a possible indicator of future CDs [28, 39, 55–57]. Therefore, it is essential to accurately and reliably measure the MF in both apparently healthy and unhealthy populations. A test of MF must be able to assess the capacity of skeletal muscle to adjust its utilization of substrate pathways in response to a metabolic challenge. However, there is no standardised method for assessing MF, with a variety of approaches available including glucose and insulin infusions, dietary challenges, epinephrine infusions, sleep challenges and more recently sub-maximal and maximal exercise [17, 38, 41, 56, 58].

### Exercise and the Assessment of Metabolic Flexibility

Exercise provides an ideal challenge to the metabolic environment of skeletal muscle where fat and CHO use within the mitochondria change depending on the stress placed on the body. The most common method of assessing the metabolic response to exercise is through indirect calorimetry [59]. Fat and CHO oxidation are calculated using a stoichiometric equation to determine maximal fat oxidation (MFO) and the exercise intensity eliciting MFO (Fat_max_) [60]. A reliance on CHO oxidation or conversely low MFO values at low-moderate exercise intensities indicates poor MF. However, Fat_max_ values reported in the literature range from as low as 20% up to 80% [61–63]. High inter-individual variability and discrepant findings among studies have further added to the difficulty in standardizing an exercise test for MF [64].

### Confounding Factors Influencing Metabolic Flexibility

There are a number of factors that can influence substrate utilization and therefore MF assessment during exercise. These factors can be separated into either controllable or non-controllable factors and are briefly discussed below.

#### Non-controllable

##### Sex

Most studies have reported significant differences in fat oxidation between males and females with differences further increased when values are normalised relative to body mass and composition [65].

##### Menopause

The loss of female sex hormones in post-menopausal women may influence fat oxidation during exercise [66]. In addition, hormone replacement therapy has been shown to increase mitochondrial content and respiratory capacity in postmenopausal women [67].

##### Genetics

Polymorphism of several genes has been shown to directly influence the rate of fatty acid oxidation [68].

##### Age

Due to a range of causes (loss of muscle mass, hormones etc.), older individuals have shown a reduced capacity to oxidise fat at the same absolute and relative exercise intensities as younger individuals [69].

##### Training status

Aerobically trained individuals have a significantly greater MFO and Fat_max_ compared to untrained and/or sedentary individuals [55].

##### Autonomic function

Recent evidence has shown that autonomic function assessed by heart rate variability enhances metabolic flexibility [70].

##### Body composition

There are conflicting views on the effect of body composition on fat oxidation with some [71] but not others [72] reporting fat mass and muscle mass influence oxidation rates.

##### Pathological condition

Many chronic diseases and disorders have a profound effect on the body’s ability to utilize fats and carbohydrates as a fuel source [58].

##### Chronic nutritional intake

Modifications in long-term (> 3 months) carbohydrate and protein intake can result in significant changes in MFO during submaximal exercise [63].

To minimise variation and possible sources of error from non-controllable factors it is suggested that participants with similar characteristics are grouped together for the assessment of MFO and CHO. Any comparisons among different groups must therefore be treated with caution.

#### Controllable

##### Environment

Increases in environmental temperature can lead to reductions in fat oxidation and increases in carbohydrate oxidation at a given exercise intensity [73].

##### Acute nutritional intake

The length of the fasting period before the exercise test may influence the subsequent values of MFO and CHO oxidation [74].

##### Time of day

Diurnal variation in substrate utilization has been reported during exercise with increased MFO in the evening compared with early morning [75].

##### Mode of exercise

The majority of studies have compared treadmill vs. cycle ergometry, with no clear consensus on whether there are any differences in MFO and CHO oxidation between the two modalities [59].

##### Stoichiometric equations

Different stoichiometric equations may produce up to a 7% variation in MFO but no difference in Fat_max_ so the same equation should be used for a within study design to minimise any possible variation in calculated values [76].

The standardisation of the controllable conditions and equipment will ensure possible sources of variation are reduced resulting in more accurate and reliable data.

### What is the Protocol for Exercise Testing of Metabolic Flexibility?

Since the original protocol for determining MFO was validated over 20 years ago [77] there have been a variety of different protocols applied across a range of studies involving athletic, healthy, and clinical populations. What is clear from these studies is that there is no “one size fits all” protocol that can be used across a range of populations [64]. In particular the non-controllable factors mentioned previously may dictate the type of exercise protocol that is implemented. For example, a recent study that compared athletic, sedentary, and clinical populations using the same protocol, may have commenced the testing the sedentary and clinical groups at workloads above MFO [78]. The main components of a protocol and how these can be adjusted to suit different populations are briefly discussed below.

#### Protocol Components

##### Starting workload

Determined by the general health and aerobic capacity of the individual with sedentary and clinical groups requiring a low initial workload to ensure the first workloads are below the MFO. It has been reported that very sedentary and some clinical populations have very poor fat oxidation at rest [40] so perhaps resting fat oxidation could help determine the first workload. For healthy and athletic populations it is suggested that if a VO_2_ max test has been completed beforehand, the initial workload could start below the graded exercise test where the MFO often occurs [79].

##### Step duration and analysis

Each step needs to be of sufficient duration to allow steady state to be achieved, yet not too long so that it affects substrate utilization. Although 3 min stages have been used with athletes [77], individuals with low aerobic capacity may require more time in each stage to reach steady state [80]. To determine the appropriate step duration, Chrzanowski-Smith and colleagues [81] compared various stage durations (4, 6, and 8 min) in sedentary females and reported that 4-minute stages were appropriate for the assessment of MFO. It has also been suggested to analyse the last 60 s of data for each 4-minute stage [59].

##### Step intensity

Step intensity is arguably the most important component of the exercise protocol as exercise intensity has a direct effect on muscle energy turnover and substrate selection [82]. The increments in step intensity can also be difficult to determine. Many small increments in intensity may prolong the test (see below) whereas large increases in intensity for each step may cause the exact point of MFO to be missed. Similar to the starting workload, step intensity will be determined by an individual’s health and aerobic fitness. For example, increments of 35 W have been used with athletic populations [77] compared to 15 W increments with untrained overweight women [83].

##### Test duration and termination

Test duration is a combination of the step duration and the number of steps in the MFO protocol. Currently there is no consensus about the ideal test duration although we do recommend a test duration of no more than ~ 24 min (6 × 4 min stages) for clinical and sedentary individuals as a longer test duration may affect substrate utilisation. A longer test duration may be used for aerobically trained athletes due to their training adaptations. Generally, most studies have stopped MFO assessment when the respiratory exchange ratio (RER) reaches 1.0 with Amaro-Gahete and colleagues [84] recently suggesting an RER of 0.95 is sufficient.

### Lactate as a Marker of Metabolic Flexibility

Lactate is a metabolite produced from CHO oxidation and the subsequent breakdown of glucose and glycogen in the glycolytic pathway. As such, a rise in lactate concentration observed during incremental exercise is primarily due to increases in CHO catabolism to meet the increased metabolic demands placed on the working muscle [85]. Therefore, increases in lactate concentration could be used as a marker of increased CHO oxidation. This was clearly shown in a recent study by San-Millan and Brooks [78] who reported an inverse relationship between blood lactate and fat oxidation and suggested that assessing blood lactate could be an effective way to indirectly assess MF.

With this in mind we recommend that increases in lactate concentration during the MF assessment could provide the cut-off point to terminate the test. In support of this concept, increasing CHO oxidation and lactate concentration have been shown to directly down-regulate fat oxidation during exercise [55] and a higher lactate concentration also indicates an increase in non-metabolic carbon dioxide production which has been shown to overestimate CHO oxidation [86, 87]. Our suggestion is to terminate the test when blood lactate measurements (to confirm the rise in lactate) taken at the end of two consecutive workload stages are 2 mmol/L above resting values. The resultant lactate concentration is similar to values reported to occur at the point of MFO in some [78, 88] but not all studies [89]. However further investigation is needed to determine if lactate concentration can be used as a method of exercise termination during MF assessment.

### Maximal or Sub-maximal Exercise Protocol

Original investigations to determine a suitable protocol for the assessment of fat and CHO oxidation used workloads that were based on an estimated VO_2_ max [90] or results from an actual VO_2_ max test [77]. Although Achten and colleagues [77] found no significant difference in MFO when comparing their VO_2_ max test with subsequent sub-maximal tests, others have reported significant differences in MFO from maximal and sub-maximal exercise tests [91]. Today both maximal and sub-maximal tests are used across a number of studies, but it is our contention that the assessment of MFO and Fat_max_ should be a separate sub-maximal exercise test.

#### The case for a “stand-alone” Fatmax test

A sub-maximal exercise test taking less than 30 min, with low to moderate effort may be all that is required to determine metabolic health. The Fat_max_ test not only provides information on substrate utilization and MF but, of equal importance, the absolute intensity and heart rate at MFO. These variables can then be used to prescribe exercise at the appropriate intensity for both athletic, non-athletic, and clinical populations. This method of exercise prescription has been successfully used on a variety of populations with positive outcomes in health and performance (for a comprehensive review see [55]).

A VO_2_ max test as part of or separate to the Fat_max_ test may not be necessary to determine MF and metabolic health. While the VO_2_ max test is considered the “gold standard” of cardiovascular health and fitness [92], the Fat_max_ test may be a more appropriate assessment of mitochondrial function and metabolic health [28, 55]. Furthermore, the VO_2_ max test requires significant motivation and maximal effort to reach volitional exhaustion. This is often associated with considerable pain and discomfort for the participant. The timing of the VO_2_ max test for athletes also needs to be carefully considered so as not to impair performance on subsequent days [93]. Additionally, there are safety concerns for elderly and other at risk clinical populations [94]. Finally, recent evidence indicates that mitochondrial oxidative capacity may be increased without changes in VO_2_ max after a period of endurance training [95].

## Conclusion and Future Direction

In conclusion it appears even among homogeneous groups that fat oxidation is a very individual response with large variations in MFO and Fat_max_ with recent data reporting predicted heart rates being substantially different from actual heart rate measurements during fat oxidation tests [96]. Mathematical modelling has also failed to accurately predict substrate oxidation during submaximal cycling and treadmill exercise [97, 98]. Further work is therefore required to determine the most appropriate protocol for the assessment of fat oxidation and MF. Regardless of the current ambiguity, the Fat_max_ test provides the opportunity of an easy, time efficient, relatively non-invasive measure of MF and mitochondrial health.

## Data Availability

Data sharing is not applicable to this article as no datasets were generated or analysed during the current study.

## Abbreviations

CD: Chronic disease
CHO: Carbohydrate
CVD: Cardiovascular disease
DAG: Diacylglycerol
Fat_max_: Exercise Intensity eliciting MFO
IR: Insulin resistance
MFO: Maximal fat oxidation
MF: Metabolic flexibility
MetS: Metabolic syndrome
MD: Mitochondrial dysfunction
VO_2_ max: Maximal oxygen uptake
